# Synergistic Antimicrobial and Antibiofilm Activity of Nitroxoline in Combination with Hydroquinone Against Uropathogenic *Enterococcus faecalis*

**DOI:** 10.3390/antibiotics15040333

**Published:** 2026-03-25

**Authors:** Davorka Repac Antić, Silvestar Mežnarić, Marko Kolenc, Irena Brčić Karačonji, Ivana Gobin

**Affiliations:** 1 Department of Microbiology and Parasitology, Faculty of Medicine, University of Rijeka, 51000 Rijeka, Croatia; davorkaar@uniri.hr; 2 Department of Clinical Microbiology, Clinical Hospital Center Rijeka, 51000 Rijeka, Croatia; 3 Department of Basic and Clinical Pharmacology with Toxicology, Faculty of Medicine, University of Rijeka, 51000 Rijeka, Croatia; silvestar.meznaric@medri.uniri.hr; 4 Institute of Microbiology and Immunology, Faculty of Medicine, University of Ljubljana, 1000 Ljubljana, Slovenia; marko.kolenc@mf.uni-lj.si; 5 Division of Toxicology, Institute for Medical Research and Occupational Health, 10000 Zagreb, Croatia; ibrcic@imi.hr; 6 Department of Basic Medical Sciences, Faculty of Health Studies, University of Rijeka, 51000 Rijeka, Croatia; 7 Teaching Institute of Public Health of Primorje-Gorski Kotar County, 51000 Rijeka, Croatia

**Keywords:** nitroxoline, hydroquinone, UTI, *Enterococcus*

## Abstract

**Background:** *Enterococcus faecalis* is a major cause of complicated urinary tract infections (UTIs), characterized by intrinsic resistance and pronounced biofilm formation. Nitroxoline (NTX), a metal-chelating uroantiseptic, accumulates in urine and exhibits antibiofilm activity. Hydroquinone (HQ), the active urinary metabolite of arbutin-containing herbal preparations, is also excreted into urine and may contribute to antimicrobial activity in situ. This study investigated the antimicrobial and antibiofilm effects of NTX and HQ, individually and in combination, against uropathogenic *E. faecalis* isolates. **Methods:** Minimum inhibitory (MIC), bactericidal (MBC), and anti-adhesion (MAC) concentrations were determined using broth microdilution. Interaction was assessed by the checkerboard method and expressed as the fractional inhibitory concentration index (FICI). Biofilm inhibition was quantified by colony-forming unit (CFU) enumeration following exposure to subinhibitory concentrations. Ultrastructural alterations of *E. faecalis* following exposure to NTX and HQ were examined by transmission electron microscopy (TEM). **Results:** NTX demonstrated MIC values ranging from 0.002–0.016 mg/mL (MIC50/MIC90: 0.004/0.008 mg/mL), while HQ exhibited MIC values of 0.78–1.56 mg/mL (MIC50/MIC90: 0.78/1.56 mg/mL). Synergistic interactions (FICI ≤ 0.5) were observed in selected isolates, with up to eightfold and sixteenfold reductions in NTX and HQ concentrations, respectively. Additive effects predominated in the remaining isolates without antagonism. The combination achieved 3–5 log_10_ reductions in adherent bacterial counts compared to untreated controls and up to 4 log_10_ reductions compared to single-agent exposure. In several strains, complete inhibition of adhesion was observed. TEM analysis revealed marked envelope disruption, cytoplasmic condensation, and structural collapse following combined treatment. **Conclusions:** Given that both NTX and HQ are active within the urinary environment, their combination may represent a pharmacologically relevant strategy targeting both bacterial growth and early biofilm establishment in enterococcal UTIs. These findings support further in vivo and pharmacokinetic investigations to evaluate the clinical applicability of this combination.

## 1. Introduction

Urinary tract infections (UTIs) remain among the most common bacterial infections worldwide and are increasingly complicated by antimicrobial resistance [1,2]. Although *Escherichia coli* predominates, *Enterococcus faecalis* is a major cause of complicated, recurrent, and healthcare-associated UTIs [1]. Enterococci combine intrinsic resistance to several antibiotic classes with the capacity to acquire additional resistance determinants and form persistent biofilms, substantially limiting therapeutic options [1,3,4]. Their reduced susceptibility to bactericidal effects of cell wall-active agents, partly due to impaired autolytic mechanisms, further contributes to treatment failure and these characteristics highlight the need to reassess existing antimicrobial agents and explore rational combination strategies [1,2,5].

Nitroxoline (5-nitro-8-hydroxyquinoline, NTX) is an established uroantiseptic historically used for uncomplicated UTIs, primarily targeting Gram-negative uropathogens. Despite its limited emphasis in current guidelines for enterococcal infections, NTX has regained attention due to its activity against multidrug-resistant bacteria and pronounced antibiofilm effects [6,7,8]. Its mechanism of action differs from that of conventional antibiotics and relies predominantly on metal chelation, interfering with essential enzymatic and metabolic pathways [7,8,9,10,11]. Given the central role of biofilm formation in enterococcal pathogenesis, NTX warrants investigation beyond its traditional Gram-negative indication. Of all antibiotic groups, 8-hydroxyquinoline derivatives are currently the only known compounds whose antibacterial activity and overall bioactivity rely almost exclusively on their chelating properties [12,13,14]. Chelation is also responsible for NTX’s unique ability to inhibit bacterial biofilm formation [8,15,16]. Plant-derived metabolites represent another source of bioactive compounds relevant to urinary tract infections. Preparations containing *Arctostaphylos uva-ursi* and *Arbutus unedo* are traditionally used as supportive therapy in UTIs [17,18]. Their principal active constituent, arbutin, is a glycosylated hydroquinone (HQ) derivative that undergoes gastrointestinal absorption and hepatic conjugation to glucuronide and sulfate forms, which are predominantly excreted in urine [19,20,21]. In the urinary tract, these conjugates may serve as precursors of free HQ, the active antimicrobial species. HQ exerts bacteriostatic and bactericidal effects through membrane destabilization and induction of oxidative stress. However, its activity against enterococci and its interaction with established uroantiseptics remain insufficiently characterized [9,17,22,23,24]. Because both NTX and HQ (derived from arbutin metabolism) may be present in the urinary environment, their combined activity could be clinically relevant. Although both compounds exhibit individual antimicrobial properties, their interaction has not been sufficiently explored and may result in synergistic, additive, or antagonistic effects that cannot be predicted from their standalone activity. The present study therefore aimed to evaluate the antimicrobial and antibiofilm activity of NTX against uropathogenic *E. faecalis* isolates and to assess its interaction with HQ, modelling a potential coexistence of conventional antibiotic therapy and plant-derived urinary metabolites in enterococcal UTIs.

## 2. Results

### 2.1. Antimicrobial Effect of Hydroquinone on E. faecalis

The antimicrobial and anti-adhesion activities of NTX and HQ against the selected *E. faecalis* isolates are summarized in Table 1.

Among clinical *E. faecalis* isolates (n = 28), NTX minimum inhibitory concentration (MIC) values ranged from 0.002 to 0.016 mg/mL, with MIC50 and MIC90 values of 0.004 and 0.008 mg/mL, respectively (Table 1). Minimum bactericidal concentration (MBC) values ranged from 0.032 to >0.064 mg/mL (MBC50/MBC90: 0.032/>0.064 mg/mL), while minimum anti-adhesion concentration (MAC) values ranged from 0.002 to 0.008 mg/mL (MAC50/MAC90: 0.004/0.004 mg/mL). HQ exhibited MIC values of 0.78–1.56 mg/mL (MIC50/MIC90: 0.78/1.56 mg/mL), MBC values of 1.56–3.12 mg/mL (MBC50/MBC90: 1.56/3.12 mg/mL), and MAC values of 0.78–1.56 mg/mL (MAC50/MAC90: 0.78/1.56 mg/mL) (Table 1). Notably, NTX exhibited MAC values that were consistently lower than or equal to its MBC values, suggesting a stable anti-adhesion effect at concentrations below those required for bactericidal activity.

### 2.2. Interactions of Nitroxoline and Hydroquinone Assessed by the Checkerboard Assay

The interaction between NTX and HQ was evaluated using the checkerboard microdilution method. Based on their individual MIC values, the tested *E. faecalis* isolates were categorized into five groups (Table 2). The interaction was interpreted according to the fractional inhibitory concentration index (FICI), where synergy was defined as FICI ≤ 0.5, additivity as 0.5 < FICI ≤ 4.0, and antagonism as FICI > 4.0 (Table 2, Table 3 and Table 4).

Synergistic interactions between NTX and HQ were observed in isolates with FICI values ranging from 0.375 to 0.5. No antagonistic effects were detected.

The interaction between NTX and HQ demonstrated synergistic activity (FICI ≤ 0.5) across multiple *E. faecalis* isolate groups (Table 3). Synergy was observed at different baseline MIC profiles, with FICI values ranging from 0.311 to 0.50. In isolates with individual MICs of 0.004/0.78 mg/mL (NTX/HQ), the combination reduced both agents fourfold (FICI = 0.50). Enhanced synergistic effects were observed in selected isolates, including E95 and the group comprising E41 and E45, where FICI values ranged from 0.375 to 0.50, corresponding to an up to eightfold reduction of NTX and fourfold reduction of HQ. Notably, the strongest synergistic interaction (FICI 0.311–0.50) was detected in isolates E7 and E40, as well as in the group including E72, E40, and ATCC 29212, where HQ concentrations were reduced up to sixteenfold. Similarly, isolates with higher baseline MIC values (0.016/0.78 mg/mL) demonstrated consistent synergy, with a fourfold reduction of NTX and an up to eightfold reduction of HQ.

Overall, synergy was observed across diverse susceptibility profiles, indicating that the NTX–HQ combination enhances antimicrobial activity independently of the baseline MIC values. No antagonistic interactions were detected.

Additive antimicrobial effects (0.5 < FICI ≤ 4.0) were observed across the majority of the tested *E. faecalis* isolate groups (Table 4). FICI values ranged from 0.56 to 0.75, indicating consistent enhancement of activity without antagonism. In isolates with baseline MIC values of 0.004/0.78 mg/mL (NTX/HQ), combination therapy resulted in a two- to fourfold reduction of NTX and an up to eightfold reduction of HQ concentrations. Similarly, isolates with individual MICs of 0.004/1.56 mg/mL demonstrated an up to fourfold reduction of NTX and an up to sixteenfold reduction of HQ. For isolates with higher baseline nitroxoline MICs (0.008–0.016 mg/mL), additive effects were maintained, with NTX reductions ranging from two- to sixteenfold and HQ reductions from two- to sixteenfold. Notably, even in strains with elevated baseline MICs, the combination consistently lowered effective concentrations of both agents. Overall, additive interactions predominated across diverse susceptibility profiles, while no antagonistic effects were detected.

### 2.3. Inhibition of Biofilm Formation by Selected Concentrations of Nitroxoline and Hydroquinone

**Figure 1:**
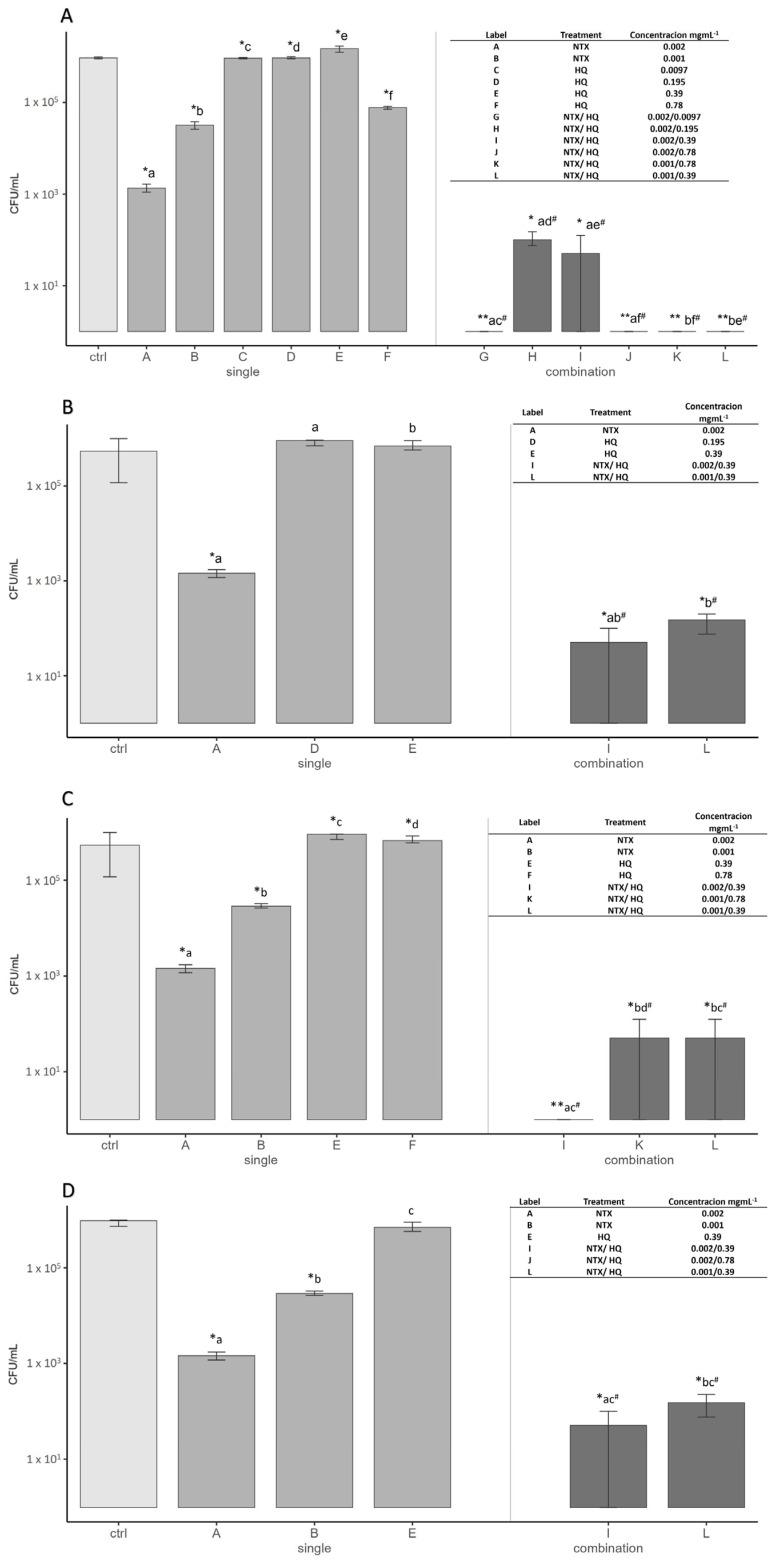
Inhibition of biofilm formation by nitroxoline (NTX) and hydroquinone (HQ), applied individually and in combination. (**A**) *Enterococcus faecalis* ATCC 29212; (**B**) clinical isolate E1; (**C**) clinical isolate E16; (**D**) clinical isolate E55. Bacterial adhesion to polystyrene was quantified as colony-forming unit (CFUs)/mL after treatment with selected subinhibitory concentrations. Letters above the bars indicate statistical comparisons between treatments. Different letters denote statistically significant differences between groups. Labels A–F represent individual NTX or HQ treatments, while labels G–L represent the corresponding NTX–HQ combinations at identical concentrations as shown in the tables within each panel Lowercase letters denote pairwise comparisons between single treatments and their corresponding combinations sharing at least one compound in the same concentration; hashtag (#) by the lowercase letters denote statistical significance (*p* < 0.05). Data are presented as mean ± SD. Statistical significance is indicated as follows: * *p* < 0.05; ** *p* < 0.01.

To evaluate the antibiofilm potential of NTX and HQ, the number of adherent bacteria was quantified following exposure to selected subinhibitory concentrations applied individually and in combination. The tested isolates included *E. faecalis* ATCC 29212, E1, E16, and E55 (Figure 1). This experimental design allowed a direct comparison of the effects of each compound alone with those of their combined exposure at identical concentrations, enabling assessment of their potential interaction in the inhibition of biofilm formation.

In all of the tested strains, combined treatment resulted in a significantly greater reduction in adhered bacterial cells compared to the same concentrations applied individually. While exposure to NTX or HQ alone led to partial inhibition of adhesion, their combination produced a marked decrease in colony-forming unit (CFU) counts, reducing bacterial load from approximately 10^5^–10^6^ CFU/mL to 10^1^–10^2^ CFU/mL. This corresponded to an overall reduction of 3–5 log_10_ units compared to the untreated control and an additional decrease of 2–4 log_10_ CFU/mL relative to single-agent treatments (Figure 1). For the reference strain *E. faecalis* ATCC 29212, several NTX–HQ combinations resulted in the complete inhibition of bacterial adhesion. Similar trends were observed for the clinical isolates, where combined treatment consistently reduced CFU counts by several orders of magnitude compared with the corresponding single-agent exposures. These findings further supported the interaction between NTX and HQ, as the combined exposure produced substantially greater inhibition of bacterial adhesion than either compound applied individually.

### 2.4. Effect of Nitroxoline and Hydroquinone Combination on Bacterial Cell Morphology Using Transmission Electron Microscopy

**Figure 2:**
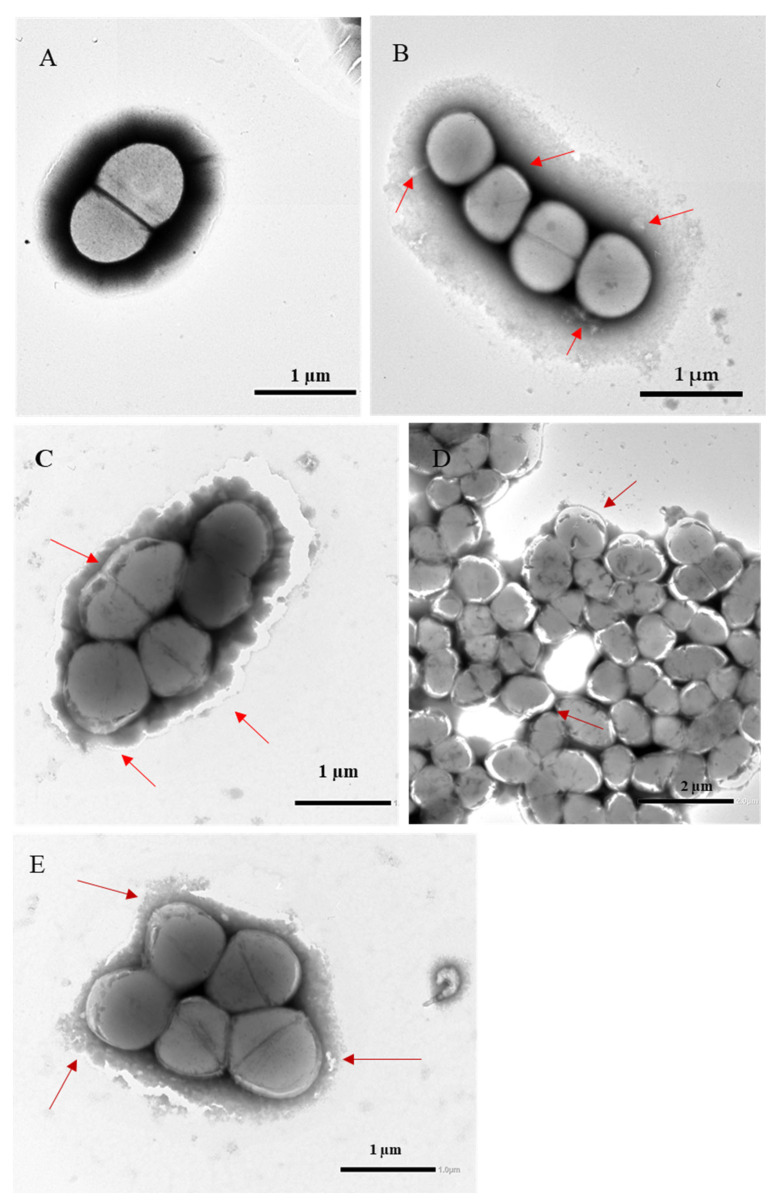
Ultrastructural alterations of *Enterococcus faecalis* ATCC 29212 following exposure to nitroxoline (NTX), hydroquinone (HQ), and their combination. (**A**) Cells were treated with NTX and HQ individually at their MIC concentrations and with the NTX–HQ combination (0.002/0.078 mg/mL) derived from the checkerboard assay. Untreated control cells exhibiting preserved spherical morphology, intact cell wall and cytoplasmic membrane, and homogeneous intracellular electron density. (**B**) Cells exposed to NTX showing moderate envelope irregularities and early membrane alterations. (**C**) Cells treated with HQ demonstrating membrane destabilization, pericellular diffuse material, and partial cytoplasmic condensation. (**D**,**E**) Cells exposed to the NTX–HQ combination revealing extensive ultrastructural disruption, including envelope disorganization, cytoplasmic condensation, structural collapse, and accumulation of extracellular amorphous material. Red arrows indicate areas of membrane discontinuity and envelope damage. Scale bars: 1 µm (**A**–**C**,**E**); 2 µm (**D**).

For transmission electron microscopy (TEM) analysis, bacterial cells were exposed to NTX and HQ at their respective MIC concentrations when tested individually. For the combination experiment, cells were treated with the NTX–HQ concentrations identified in the checkerboard assay (NTX/HQ: 0.002/0.078 mg/mL). TEM analysis revealed progressive ultrastructural alterations in *E. faecalis* following exposure to individual agents and their combination (Figure 2). Control cells (A) displayed typical coccoid morphology with smooth, continuous cell walls, clearly defined cytoplasmic membranes, and uniform electron-dense cytoplasm, indicating preserved structural integrity. Exposure to NTX alone (B) resulted in moderate structural alterations, including slight irregularities of the cell envelope and localized membrane detachment, while overall cellular architecture remained largely preserved. HQ treatment (C) induced more pronounced pericellular changes, characterized by membrane destabilization, diffuse extracellular material, and areas of cytoplasmic condensation, suggesting early envelope compromise. In contrast, the combined NTX–HQ treatment (D, E) produced extensive structural damage. Cells exhibited irregular thickening and discontinuity of the cell wall, loss of membrane integrity, cytoplasmic shrinkage, and partial structural collapse. Clusters of cocci demonstrated blurred intercellular boundaries and accumulation of amorphous extracellular material, indicative of leakage of intracellular components. The combined exposure resulted in markedly greater disruption than either agent alone.

## 3. Discussion

HQ represents the active urinary metabolite of arbutin-containing herbal preparations traditionally used in the management of UTIs [17,25,26]. Following oral administration, arbutin is metabolized and excreted predominantly as HQ glucuronide and sulfate conjugates [17,18,19,27], which may release active HQ in the urinary environment. The presence of *β*-glucosidase activity in *E. faecalis* further supports the potential for local HQ generation in situ [6], providing a biologically plausible basis for its antimicrobial activity in enterococcal UTIs. Therapeutic efficacy is therefore dependent on achieving sufficient urinary concentrations of HQ equivalents [26].

In the present study, HQ demonstrated moderate but consistent antimicrobial activity against clinical *E. faecalis* isolates, with MIC values predominantly at 0.78 mg/mL and MBC values at 1.56 mg/mL. The MAC value corresponded to the MIC, indicating that growth inhibition was accompanied by suppression of initial surface colonization. Similar in vitro antibacterial effects of arbutin and HQ have been previously reported against uropathogens [17,27]. NTX exhibited lower MIC values and maintained previously reported antibiofilm activity [8,15]. Its antimicrobial mechanism is primarily attributed to metal chelation, particularly of Mg^2+^ and Mn^2+^, which are essential cofactors in bacterial enzymatic processes [21]. Pelletier et al. demonstrated that the activity of NTX is reduced in the presence of excess Mg^2+^ and Mn^2+^, supporting chelation as the primary mechanism [21]. Additionally, 8-hydroxyquinoline derivatives have been shown to inhibit RNA polymerase activity via metal chelation in yeast models [25], suggesting indirect interference with essential cellular processes. NTX-mediated chelation of iron and zinc has also been implicated in biofilm inhibition through interference with metal-dependent regulatory pathways [27].

The most relevant finding of this study is the enhanced antimicrobial and antibiofilm activity observed when NTX and HQ were combined. Checkerboard analysis demonstrated synergistic interactions (FICI ≤ 0.5) in selected isolates and additive effects in the majority, without evidence of antagonism. Combination therapy reduced effective concentrations of both agents by up to eight-fold for NTX and sixteenfold for HQ, suggesting pharmacodynamic potentiation. Similar potentiation effects have been described for NTX-containing combinations targeting *E. faecalis* adhesion [12]. Our antibiofilm experiments further demonstrated that the interaction between NTX and HQ extends beyond planktonic growth inhibition. Subinhibitory concentrations of NTX or HQ applied individually produced only limited reductions in bacterial adhesion, whereas the same concentrations used in combination resulted in substantially stronger inhibition of biofilm formation. Notably, several combinations completely prevented adhesion of the reference strain *E. faecalis* ATCC 29212. These findings indicate that simultaneous exposure to NTX and HQ enhances antibiofilm activity and suggest a cooperative interaction between the two compounds during the early stages of biofilm establishment. They are consistent with reports demonstrating that chelation-based strategies may impair early biofilm establishment independently of bactericidal activity [15,22,28,29].

Ultrastructural analysis further supported this cooperative effect. The severity of ultrastructural damage observed under combined treatment suggested a cooperative effect on bacterial envelope stability. NTX-mediated metal chelation may interfere with membrane-associated enzymatic processes, while HQ-induced oxidative stress may further destabilize membrane structures, collectively leading to structural collapse [21]. The convergence of metal homeostasis disruption and oxidative membrane injury may underlie the enhanced structural damage observed [1,2,3,16].

NTX is an established uroantiseptic with a long clinical history and a favorable safety profile in the treatment of urinary tract infections [6,7]. HQ, although associated with cytotoxic effects at higher concentrations, is primarily present in the urinary tract as a metabolite of arbutin-containing herbal preparations traditionally used for supportive UTI therapy [17,19,26]. Therefore, the interaction between NTX and HQ may occur locally within the urinary environment, where both compounds can coexist. Such interactions may contribute to improved control of biofilm-associated infections by limiting early bacterial adhesion and biofilm establishment.

This study has several limitations that should be considered when interpreting the findings. First, all of the experiments were conducted in vitro, and therefore the observed synergistic and antibiofilm effects may not fully reflect the complex conditions of the urinary tract in vivo, including host immune responses, urine composition, and dynamic flow conditions. Second, although HQ is known to be excreted into urine following arbutin metabolism, the actual urinary concentrations achievable in clinical settings were not measured in this study. Consequently, the translational relevance of the tested concentrations requires further pharmacokinetic evaluation. Finally, the mechanistic interpretation of synergy is based on phenotypic observations and ultrastructural analysis; molecular pathways underlying the interaction between NTX and HQ were not directly investigated and warrant further study.

Taken together, these findings indicate that HQ, despite moderate intrinsic activity, can potentiate the antimicrobial and antibiofilm efficacy of NTX against uropathogenic *E. faecalis*. Given that both compounds act within the urinary environment, their combination may represent a rational adjunctive approach in enterococcal UTIs.

## 4. Materials and Methods

### 4.1. Strains, Culture Conditions, and Chemicals

A total of 29 *E. faecalis* strains were included in this study. The isolates were obtained from clinical urine samples of patients with a confirmed UTI during routine diagnostic procedures at the Clinical Microbiology Institute, Clinical Hospital Center Rijeka, Croatia. These 29 strains were selected from approximately 100 previously collected and characterized isolates, based on their phenotypic profiles, antimicrobial susceptibility patterns, and biofilm-forming capacity. In addition, the reference strain *E. faecalis* ATCC 29212 (American Type Culture Collection, Manassas, VA, USA) was included as a quality control strain. Prior to experimentation, all isolates were preserved at −80 °C in 10% glycerol stocks (Kemika, Zagreb, Croatia). For testing, bacteria were subcultured on Mueller–Hinton (MH) agar (Oxoid, Basingstoke, UK) and incubated for 24 h at 35 ± 2 °C under aerobic conditions.

NTX and HQ stock solutions were prepared according to the manufacturer’s recommendations. The HQ solution was prepared on the day of the experiment, dissolved in sterile distilled water (Aqua B. Braun, Melsungen, Germany) and stored in the dark, whereas NTX was dissolved in dimethyl sulfoxide (DMSO) (Sigma-Aldrich, Taufkirchen, Germany). The final concentration of DMSO in all of the experiments did not exceed 1% (*v*/*v*), a level considered non-inhibitory for bacterial growth. Stock solutions were aliquoted and stored at −20 °C until further use.

### 4.2. Determination of MIC, MBC, and MAC

Antimicrobial activity of HQ and NTX against clinical *E. faecalis* isolates and the reference strain *E. faecalis* ATCC 29212 was determined by broth microdilution. Results for NTX were interpreted according to current EUCAST criteria [30]. Twofold serial dilutions of HQ and NTX were prepared in 96-well microtiter plates (Vacutest Kima s.r.l., Arzergrande, Italy). The tested concentration range corresponded to 0.00025–0.256 mg/mL. Wells were inoculated with bacterial suspension adjusted to 1 × 10^6^ CFU/mL. Sterility (medium only) and growth (medium with inoculum) controls were included in triplicate. Plates were incubated for 24 h at 37 °C. The MIC was defined as the lowest concentration without visible growth.

For MBC determination, aliquots from wells without visible growth were subcultured onto MH agar and incubated for 24 h at 37 °C. MBC was defined as the lowest concentration resulting in ≥99% reduction of viable cells. MIC and MBC values were expressed in mg/mL.

MAC was defined as the lowest concentration completely preventing adhesion to polystyrene. Serial dilutions were prepared as described for MIC. After 24 h at 37 °C, supernatants were removed, wells were washed twice with sterile phosphate-buffered saline (PBS), and adherent cells were detached by sonication (Bactosonic, Bandelin, Berlin, Germany; 40 kHz, 1 min). Suspensions were plated on MH agar and incubated for 24 h at 37 °C. MAC was recorded as the lowest concentration yielding no viable adherent bacteria.

### 4.3. Evaluation of Hydroquinone-Nitroxoline Interaction by the Checkerboard Assay

The interaction between HQ and NTX against selected uropathogenic *E. faecalis* isolates was evaluated using the checkerboard microdilution method. The highest tested concentration of each compound corresponded to twice its previously determined MIC.

Twofold serial dilutions of NTX were prepared along the columns of a 96-well microtiter plate, while HQ dilutions were arranged along the rows, generating 36 different concentration combinations. Final concentrations decreased stepwise across the plate, with NTX diluted horizontally and HQ vertically. Control wells containing each agent alone and growth controls were included.

Wells were inoculated with bacterial suspension adjusted to 1 × 10^6^ CFU/mL and incubated for 24 h at 35 ± 2 °C. The MIC for each combination was determined visually as the lowest concentration showing no visible growth.

The fractional inhibitory concentration (FIC) for each agent was calculated according to the following formula:

FIC(A) = MIC(A) in combination with (B)/MIC(A)

FIC(B) = MIC(B) in combination with (A)/MIC(B).

The FIC values were then used in the formula to calculate the Fractional Inhibitory Concentration Index (FICi):

FICi = FIC(A) + FIC(B) = MIC(A)(B)/MIC(A) + MIC(B)(A)/MIC(B).

Interaction was interpreted according to the fractional inhibitory concentration index (FICI) as follows: synergy (FICI ≤ 0.5), additive or indifferent interaction (0.5 < FICI ≤ 4.0), and antagonism (FICI > 4.0) [31].

### 4.4. Inhibition of Biofilm Formation by Subinhibitory Concentrations of Hydroquinone and Nitroxoline

Biofilm inhibition was assessed using subinhibitory concentrations (1/2, 1/4, and 1/8 MIC) of NTX and HQ. All *E. faecalis* isolates were subcultured on MH agar and incubated for 18–24 h at 37 °C. Bacterial suspensions were prepared in sterile saline and adjusted to 0.5 McFarland standard (≈1.5 × 10^8^ CFU/mL), followed by dilution in MH broth to obtain a final inoculum of 1 × 10^6^ CFU/mL.

Aliquots of bacterial suspension were combined with appropriate antimicrobial dilutions to achieve the desired final concentrations (1/2, 1/4, and 1/8 MIC). A growth control containing bacteria without antimicrobial agent was included. Subsequently, 100 µL of inoculated broth and 100 µL of the corresponding antimicrobial dilution were dispensed into microtiter plate wells and incubated aerobically at 37 °C for 20–24 h.

Following incubation, non-adherent cells were removed and wells were washed twice with sterile PBS. Adherent biofilm cells were detached by sonication for 1 min in an ultrasonic bath. Serial tenfold dilutions of the recovered suspensions were plated on MH agar for viable cell enumeration.

### 4.5. Transmission Electron Microscopy Analysis

To evaluate morphological alterations induced by subinhibitory concentrations of NTX and its combination with HQ, treated *E. faecalis* cultures were processed for TEM. For TEM analysis, bacterial cells were exposed to NTX and HQ at their respective MIC concentrations when tested individually. For the combination experiment, cells were treated with the NTX–HQ concentrations identified in the checkerboard assay (NTX/HQ: 0.002/0.078 mg/mL).

A 10 µL aliquot of bacterial suspension was applied onto copper grids coated with formvar (Agar Scientific Ltd., Essex, UK) and allowed to adsorb for 2 min. Excess liquid was removed using filter paper (Whatman No. 3, Macherey-Nagel, Düren, Germany). Samples were negatively stained with 1% phosphotungstic acid (Sigma-Aldrich, St. Louis, MO, USA) for 1 min and air-dried at room temperature. The prepared grids were examined using a TEM (JEM-2100F, JEOL Ltd., Tokyo, Japan).

### 4.6. Statistical Analysis

Data processing, statistical analysis, and visualization were performed using R statistical software (version 4.4.1, R Core Team, 2024) in RStudio IDE (version 2025.09.2). Data manipulation and visualization were conducted using the tidyverse suite of packages, which includes ggplot2 for graphics, dplyr for data manipulation, and forcats for factor handling [32,33]. Logarithmic axis transformations were implemented using the scales package [32]. Due to the small sample sizes and non-normal distribution, nonparametric Mann-Whitney U tests were employed for pairwise comparisons between each treatment and control, as well as between single treatments and their respective combinations, using the rstatix package [34]. *p*-values were adjusted for multiple comparisons using the Holm correction method. The threshold for statistical significance was set at *p* = 0.05.

## 5. Conclusions

This study demonstrates that the combination of NTX and HQ enhances antimicrobial and antibiofilm activity against uropathogenic *E. faecalis*. Checkerboard analysis revealed synergistic or additive interactions without antagonism, while antibiofilm experiments showed that combined subinhibitory concentrations significantly reduced bacterial adhesion compared with individual treatments. These findings suggest that simultaneous exposure to NTX and HQ may potentiate antimicrobial activity in the urinary environment. Considering that HQ may be generated in urine following the metabolism of arbutin-containing herbal preparations, the NTX-HQ interaction may represent a pharmacologically relevant scenario during the treatment of urinary tract infections. Future studies should focus on pharmacokinetic evaluations of urinary HQ concentrations, mechanistic investigations of the observed interaction, and validation of these findings in in vivo infection models.

## Figures and Tables

**Table 1:** Antimicrobial activity of nitroxoline (NTX) and hydroquinone (HQ) against uropathogenic *Enterococcus faecalis* isolates.

Strain	NTX (mg/mL)			HQ (mg/mL)		
	MIC	MBC	MAC	MIC	MBC	MAC
E46	0.008	0.032	0.004	1.56	3.12	1.56
E61	0.016	>0.064	0.008	0.78	1.56	0.78
E34	0.002	0.032	0.002	1.56	3.12	1.56
ATCC 29212	0.008	0.032	0.004	1.56	3.12	1.56
E89	0.004	0.032	0.002	1.56	3.12	1.56
E16	0.008	>0.064	0.004	1.56	3.12	1.56
E47	0.004	0.032	0.002	1.56	3.12	1.56
E79	0.008	0.032	0.004	0.78	1.56	0.78
E39	0.004	0.032	0.002	0.78	1.56	0.78
E7	0.008	0.032	0.004	0.78	1.56	0.78
E36	0.004	0.032	0.002	0.78	1.56	0.78
E69	0.008	>0.064	0.004	0.78	1.56	0.78
E85	0.004	0.032	0.002	0.78	1.56	0.78
E10	0.004	0.032	0.002	0.78	1.56	0.78
E35	0.008	>0.064	0.004	0.78	1.56	0.78
E15	0.004	0.032	0.002	0.78	1.56	0.78
E32	0.004	0.032	0.002	1.56	3.12	1.56
E84	0.004	0.032	0.002	0.78	1.56	0.78
E95	0.004	0.032	0.002	1.56	3.12	1.56
E38	0.008	0.032	0.004	0.78	1.56	0.78
E55	0.008	0.032	0.004	0.78	1.56	0.78
E45	0.008	0.032	0.004	1.56	3.12	1.56
E72	0.008	0.032	0.004	1.56	3.12	1.56
E41	0.008	0.032	0.004	1.56	3.12	1.56
E8	0.004	0.032	0.002	0.78	1.56	0.78
E58	0.004	0.032	0.002	0.78	1.56	0.78
E1	0.004	0.032	0.002	0.78	1.56	0.78
E48	0.008	0.032	0.004	1.56	3.12	1.56
E37	0.008	>0.064	0.004	0.78	1.56	0.78

MIC—minimum inhibitory concentration; MBC—minimum bactericidal concentration; MAC—minimum anti-adhesion concentration; “>” indicates values exceeding the highest tested concentration.

**Table 2:** Distribution of *Enterococcus faecalis* isolates according to MIC combinations of nitroxoline (NTX) and hydroquinone (HQ).

Group	MIC NTX (mg/mL)	MIC HQ (mg/mL)	*E. faecalis* Strains
1	0.004	0.78	E1, E39, E58, E84, E36, E8, E15, E85
2	0.004	1.56	E47, E32, E34, E95, E89
3	0.008	0.78	E79, E10, E38, E40, E55, E7
4	0.008	1.56	E45, E41, E16, E46, E72, ATCC 29212
5	0.016	0.78	E37, E35, E61

**Table 3:** Fractional inhibitory concentration index (FICI) values of the nitroxoline (NTX)–hydroquinone (HQ) combination against *Enterococcus faecalis* isolate groups, with synergistic effects.

Isolate Group (n)	Individual MIC NTX/HQ (mg/mL)	Combination MIC NTX + HQ (mg/mL)	FICI (Range)	Fold ↓ NTX	Fold ↓ HQ
E1, E8, E36, E39, E58, E84 (6)	0.004/0.78	0.001/0.195	0.50	4×	4×
E95 (1)	0.004/1.56	0.0005/0.39–0.001/0.39	0.375–0.50	4×–8×	4×
E1, E38 (2)	0.008/0.78	0.002/0.195	0.50	4×	4×
E7, E40 (2)	0.008/0.78	0.002/0.048–0.002/0.097–0.002/0.195	0.311–0.50	4×	4×–16×
E16 (1)	0.008/1.56	0.002/0.39	0.50	4×	4×
E41, E45 (2)	0.008/1.56	0.002/0.39–0.001/0.39	0.375–0.50	4×–8×	4×
E72, E40, ATCC 29212 (3)	0.008/1.56	0.002/0.097–0.002/0.195–0.002/0.39	0.312–0.50	4×	8×–16×
E37, E35, E61 (3)	0.016/0.78	0.004/0.097–0.004/0.195	0.374–0.50	4×	4×–8×

MIC—minimum inhibitory concentration; FICI—fractional inhibitory concentration index. FIC = MIC in combination/MIC alone; FICI = FIC_NTX + FIC_HQ. Synergy defined as FICI ≤ 0.5.

**Table 4:** Additive antimicrobial activity of the nitroxoline (NTX) and hydroquinone (HQ) combination against *Enterococcus faecalis* isolate groups.

Isolate Group (n)	Individual MIC NTX/HQ (mg/mL)	Combination MIC NTX + HQ (mg/mL)	FICI (Range)	Fold ↓ NTX	Fold ↓ HQ
E1, E8, E36, E39, E58, E84 (6)	0.004/0.78	0.002/0.097–0.002/0.195–0.001/0.39	0.624–0.75	2×–4×	2×–8×
E15, E85 (2)	0.004/0.78	0.002/0.097–0.002/0.195	0.624–0.75	2×	4×–8×
E32, E47, E89, E95 (4)	0.004/1.56	0.002/0.097–0.002/0.195–0.001/0.39	0.56–0.75	2×–4×	4×–16×
E7, E40 (2)	0.008/0.78	0.004/0.048–0.004/0.097–0.004/0.195	0.561–0.75	2×	4×–16×
E10, E38 (2)	0.008/0.78	0.004/0.097–0.004/0.195	0.624–0.75	2×	4×–8×
E55, E79 (2)	0.008/0.78	0.002/0.39–0.004/0.097–0.004/0.195–0.001/0.39	0.625–0.75	2×–8×	2×–8×
E41, E45, E6, E16, E72, ATCC 29212 (6)	0.008/1.56	0.0005/0.78–0.001/0.78–0.002/0.78–0.004/0.097–0.004/0.195–0.004/0.39	0.50–0.75	2×–16×	2×–16×
E37, E35, E61 (3)	0.016/0.78	0.004/0.39	0.75	4×	2×

MIC—minimum inhibitory concentration; FICI—fractional inhibitory concentration index. FIC = MIC in combination/MIC alone; FICI = FIC_NTX + FIC_HQ. Synergy: FICI ≤ 0.5; Additive/Indifferent: 0.5 < FICI ≤ 4.0.

## Data Availability

Data are contained within the article. The raw data supporting the conclusions of this article will be made available by the authors on request.
